# Increased rates of infantile hypercalcaemia following guidelines for antenatal vitamin D3 supplementation

**DOI:** 10.1186/1687-9856-2015-S1-O42

**Published:** 2015-04-28

**Authors:** Lisa A Amato, Shihab Hameed, Kris A Neville, Wei Shern Quek, Andrea R Horvath, Chris P White, Charles F Verge, Helen J Woodhead, Jan L Walker

**Affiliations:** 1 Endocrinology, Sydney Children's Hospital, Sydney, NSW, Australia; 2 School of Women’s and Children’s Health, UNSW, Sydney, NSW, Australia; 3 SEALS Clinical Chemistry, Prince of Wales Hospital, Sydney, NSW, Australia

## 

Consultations for infantile hypercalcaemia (IIH) have increased at Sydney Children’s Hospital since guidelines for vitamin D_3_ supplementation during pregnancy were introduced in 2006. Recent nationwide shortages of low-calcium formula (LCF) suggest this problem may be widespread. CYP24A1 mutations have been identified as a potential cause of IIH.

To determine if IIH is occurring more commonly, de-identified, first-measured serum calcium from all infants <6 months (n=5796) measured in our laboratory, were grouped by years 2005-2007 (n=1516), 2008-2010 (n=1945) and 2011-2013 (n=2335). In addition, we analysed 13 infants treated by our department for idiopathic infantile hypercalcaemia (IIH) from 2011-2013.

Rates of hypercalcaemia (>2.75mmol/L) increased from 2011 (1.1% vs 1.3% vs 8.7%, χ^2^P<0.001). Rates of hypocalcaemia (<2.25mmol/L) fell steadily (42.4% vs 32.3% vs 24.8% %, χ^2^P<0.001). Twelve mothers of our 13 infants with IIH received antenatal vitamin D_3_ supplementation. One infant also received 400 units/day Vitamin D_3_ post-natally. At diagnosis, median age was 13 days (range 4-50), 77% were breast-fed, 54% were symptomatic and 25% had nephrocalcinosis. Median initial calcium was 3.00mmol/L (range 2.84-4.03) and phosphate 2.04mmol/L (1.1-3.33). PTH was not elevated (median 1.0pmol/L [<0.3-3.1]), urinary calcium: creatinine ratio not suppressed (median 2.3, [0.4-9]), 25OHVitD low-normal (median 44nmol/L [17-218]) and 1,25(OH)_2_VitD elevated (median 232pmol/L [64-720]), in keeping with an abnormality in CYP24A1. In 7/10 infants with data available, treated with LCF for median 95 days (range 25-310), median PTH rose to 17.1pmol/L ([8.2-49.3], P=0.02) with a trend to lower 25OHVit D (median 23nmol/L [<10-108], P=0.09) despite continued high-normal calcium levels (median 2.66mmol/L [2.11-2.75]).

Concurrent changes in rates of hyper and hypo-calcaemia suggest antenatal vitamin D_3_ supplementation as an aetiological factor. IIH was associated with significant morbidity, including symptomatic hypercalcaemia and nephrocalcinosis. Treatment with LCF prevented further symptomatic hypercalcaemia, but resulted in elevated PTH. The biochemistry of our patients with IIH raises variations in Vitamin D metabolism or calcium set-point as potential associated factors.

